# Expression of Longevity Genes Induced by a Low-Dose Fluvastatin and Valsartan Combination with the Potential to Prevent/Treat “Aging-Related Disorders”

**DOI:** 10.3390/ijms20081844

**Published:** 2019-04-14

**Authors:** Miodrag Janić, Mojca Lunder, Srdjan Novaković, Petra Škerl, Mišo Šabovič

**Affiliations:** 1 Department of Vascular Diseases, Ljubljana University Medical Centre, Zaloška cesta 7, SI-1000 Ljubljana, Slovenia; miodrag.janic@kclj.si; 2 Department of Endocrinology, Diabetes and Metabolic Diseases, Ljubljana University Medical Centre, Zaloška cesta 7, SI-1000 Ljubljana, Slovenia; mojca.lunder@kclj.si; 3 Department of Molecular Diagnostics, Institute of Oncology Ljubljana, SI-1000 Ljubljana, Slovenia; snovakovic@onko-i.si (S.N.); pskerl@onko-i.si (P.Š.)

**Keywords:** aging-related disorders, longevity genes, arterial aging, low-dose fluvastatin and valsartan combination

## Abstract

The incidence of aging-related disorders may be decreased through strategies influencing the expression of longevity genes. Although numerous approaches have been suggested, no effective, safe, and easily applicable approach is yet available. Efficacy of low-dose fluvastatin and valsartan, separately or in combination, on the expression of the longevity genes in middle-aged males, was assessed. Stored blood samples from 130 apparently healthy middle-aged males treated with fluvastatin (10 mg daily), valsartan (20 mg daily), fluvastatin-valsartan combination (10 and 20 mg, respectively), and placebo (control) were analyzed. They were taken before and after 30 days of treatment and, additionally, five months after treatment discontinuation. The expression of the following longevity genes was assessed: *SIRT1*, *PRKAA*, *KLOTHO*, *NFE2L2*, *mTOR*, and *NF-κB*. Treatment with fluvastatin and valsartan in combination significantly increased the expression of *SIRT1* (1.8-fold; *p* < 0.0001), *PRKAA* (1.5-fold; *p* = 0.262) and *KLOTHO* (1.7-fold; *p* < 0.0001), but not *NFE2L2*, *mTOR* and *NF-κB*. Both fluvastatin and valsartan alone significantly, but to a lesser extent, increased the expression of *SIRT1*, and did not influence the expression of other genes. Five months after treatment discontinuation, genes expression decreased to the basal levels. In addition, analysis with previously obtained results revealed significant correlation between *SIRT1* and both increased telomerase activity and improved arterial wall characteristics. We showed that low-dose fluvastatin and valsartan, separately and in combination, substantially increase expression of *SIRT1*, *PRKAA*, and *KLOTHO* genes, which may be attributed to their so far unreported pleiotropic beneficial effects. This approach could be used for prevention of ageing (and longevity genes)–related disorders.

## 1. Introduction

An aging population, along with increased life expectancy and the prevalence of associated chronic diseases, has become an important medical and economic issue. Consequently, the burden of so-called “aging-related disorders,” such as associated cardiovascular diseases, degenerative diseases of the central nervous system, and malignant diseases, is also increasing [1,2]. These diseases represent one of the leading problems of the healthcare systems in developed countries around the globe. Effective strategies for their prevention are therefore needed. Aging-related disorders have been found to be causally associated with the altered expression of aging-related or so-called longevity genes [3]. In addition, telomere length per se [4], as well as telomerase expression [5], are also associated with aging-related disorders. In a narrower context, it seems logical that these genes represent mechanistic intracellular targets that could be altered to change the intracellular pro-aging milieu. It is expected that with the induction of expression of protective and suppression of expression of harmful genes, a new rejuvenated cellular phenotype could be reached that could influence the occurrence and course of aging-related disorders. In summary, so-called rejuvenating strategies, focused on modifying such genes’ expression, could possibly importantly impact the prevalence of aging-related disorders [3].

In our prior studies, we explored the functional and structural characteristics of the arterial wall, some of which are also characteristic for arterial aging, and were particularly interested in the improvement of altered characteristics of the arterial wall by low doses of fluvastatin and valsartan [6]. We found significant improvement of arterial wall characteristics after 30 days of treatment in middle-aged males, with the beneficial effect slowly declining within nine months of treatment discontinuation [7,8,9]. Importantly, for age-related changes, the improvement of arterial wall characteristics was associated with increased telomerase expression and reduced inflammation as well as oxidative stress parameters [10,11]. Both decreased telomerase expression along with decreased telomeres’ length and increased activation of inflammation and oxidative stress are characteristic of aging, arterial aging, and aging-related disorders. Therefore, we hypothesized that “anti-aging” or “rejuvenation” effects could be achieved through treatment impacting the longevity genes. This impact should have the capacity to influence “aging-related” disorders. Considering our previous results, this approach could be particularly effective for decreasing aging-related changes of the arterial system.

In the present study, we explored the efficacy of an approach consisting of (short-term) treatment with low-dose fluvastatin and valsartan alone or in combination on the expression of longevity genes in middle-aged males who had already (sub-clinically) impaired functional and structural arterial wall characteristics which could at least partially be attributed to the initial processes of aging.

## 2. Results

### 2.1. Expression of Longevity Genes

**Figure 1:**
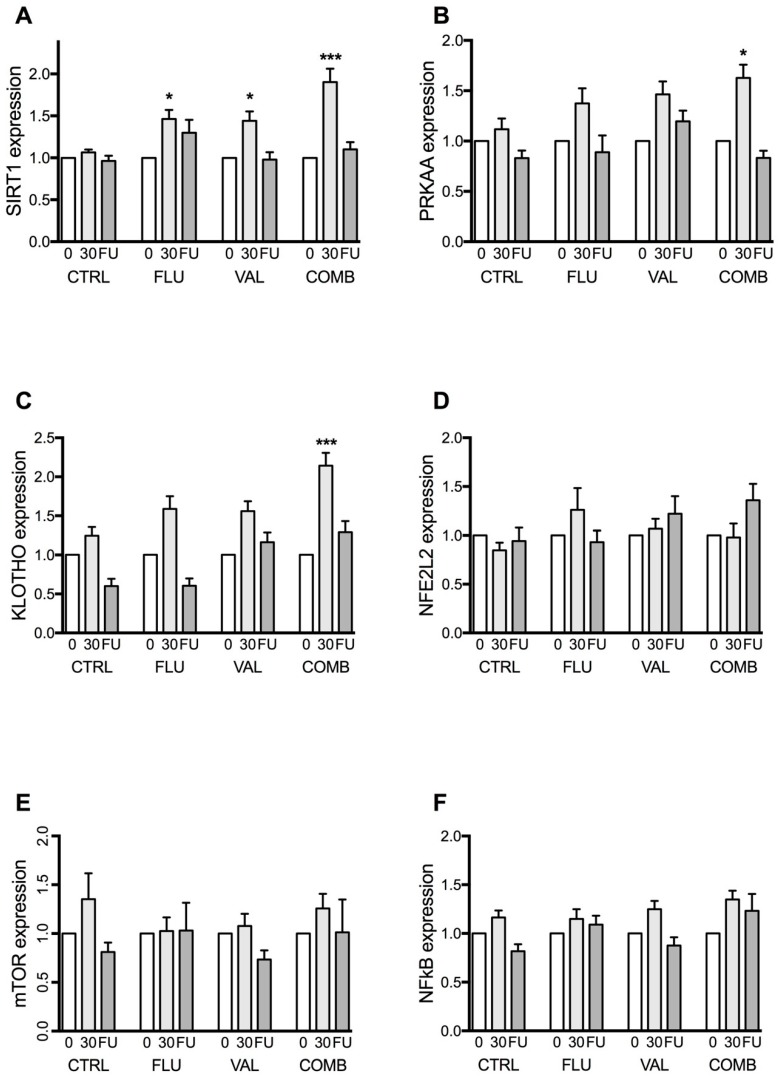
The expression of longevity genes: (**A**) *SIRT1*, (**B**) *PRKAA*, (**C**) *KLOTHO*, (**D**) *NFE2L2*, (**E**) *mTOR*, and (**F**) *NF-κB* in placebo (CTRL), low-dose fluvastatin (FLU), low-dose valsartan (VAL), or low-dose fluvastatin and valsartan combination (COMB). “0” represents the time before treatment, “30” represents the end of treatment, i.e., after 30 days (30) and “FU” represents five months after treatment discontinuation. Values are presented as means ± SEM. Presented *p* values are after Benjamini-Hochberg false discovery rate (FDR) correction, significance threshold set at *p* < 0.05. * signifies *p* < 0.05 and *** *p* < 0.001, vs. control group. *SIRT1*—sirtuin 1 gene; *PRKAA*—5′-AMP-activated protein kinase catalytic subunit α-2 gene; *NFE2L2*—nuclear factor (erythroid-derived 2)-like 2 gene; *mTOR*—mechanistic target of rapamycin (mTOR) gene; *NF-κB1*—nuclear factor κB gene.

We analyzed the expression of longevity genes in the treatment and control groups. At the beginning of the study, there was no difference in longevity genes expression in the four different groups (low-dose fluvastatin, low-dose valsartan, low-dose fluvastatin and valsartan combination, and the control group). Differences between the groups were only observed after 30 days of treatment. Five months after treatment discontinuation, the expression of longevity genes in all treatment groups decreased almost to initial values. In the control group, the expression of longevity genes did not change during the study (Figure 1A–F).

#### 2.1.1. Sirtuin 1 (*SIRT1*) Gene Expression

Both low-dose fluvastatin and valsartan separately increased the expression of the *SIRT1* gene after 30 days of treatment up to 1.4-fold (*p* = 0.0165 and *p* = 0.0229, respectively), while their low-dose combination increased its expression up to 1.8-fold (*p* < 0.0001) compared to the control group. Five months after treatment discontinuation, no significant effects of the treatment on the *SIRT1* gene expression were observed (Figure 1A).

#### 2.1.2. 5’-AMP-Activated Protein Kinase Catalytic Subunit α-2 (*PRKAA*) Gene Expression

After 30 days of treatment, only the low-dose fluvastatin and valsartan combination significantly increased the expression of the *PRKAA* gene, up to 1.5-fold (*p* = 0.0262) compared to the control group. Separate drugs had no influence on the *PRKAA* gene expression (Figure 1B).

#### 2.1.3. *KLOTHO* Gene Expression

After 30 days of treatment, only the low-dose fluvastatin and valsartan combination significantly increased the expression of the *KLOTHO* gene, up to 1.7-fold (*p* < 0.0001) compared to the control group. Separate drugs had no influence on the *KLOTHO* gene expression (Figure 1C).

#### 2.1.4. Nuclear Factor (Erythroid-Derived 2)-Like 2 Gene Expression (*NFE2L2*)

No significant changes of the *NFE2L2* gene expression were observed in any of the study groups (Figure 1D).

#### 2.1.5. Mechanistic Target of Rapamycin (*mTOR*) Gene Expression

No significant changes were observed in the *mTOR* gene expression in any of the study groups (Figure 1E).

#### 2.1.6. Nuclear Factor κB (*NF-κB*) Gene Expression

No statistically significant changes in the expression of the *NF-κB* gene were observed either (Figure 1F).

### 2.2. Correlations between the Expression of Longevity Genes, Telomerase Activity and Arterial Wall Properties

The correlations between longevity genes expression and previously described telomerase activity and arterial wall properties [7,8,9,10], all measured after 30 days of treatment, were calculated. In the separate, low-dose fluvastatin and valsartan groups, the expression of the *SIRT1* gene positively correlated with telomerase activity (*r* = 0.42; *p* = 0.04 and *r* = 0.39; *p* = 0.03, respectively). Importantly, in the low-dose combination group, the expression of the *SIRT1* gene positively correlated with telomerase activity (*r* = 0.62; *p* = 0.01) and with brachial artery flow-mediated dilation (FMD) (*r* = 0.52; *p* = 0.05) while negatively correlated with carotid artery beta stiffness (*r* = −0.45; *p* = 0.02) and pulse wave velocity (PWV) (*r* = −0.56; *p* = 0.05).

## 3. Discussion

In the present study, we showed that low-dose fluvastatin and valsartan in combination significantly increased the expression of several important longevity genes (*SIRT1, PRKAA, KLOTHO*). Moreover, these changes correlated with an improvement of functional and structural arterial wall characteristics as well as with telomerase activity, both assessed previously [7,8,9,10]. Overall, the results revealed increased expression of several longevity genes that seems to be causally associated with increased telomerase activity and improvement of arterial wall (initial aging-related) characteristics. The results are very promising and indicate that our relatively simple but innovative approach could have potential efficacy as a “rejuvenating agent,” particularly in the efforts to decrease the occurrence of aging-related disorders.

The present study was designed as a two-part study: The first part comprised the measurement of longevity genes in relation to treatment with low-dose fluvastatin and valsartan, and the second part comprised of a correlation analysis with previously measured relevant parameters (telomerase activity and functional/structural arterial wall characteristics). The studied population was a group of middle-aged males with already present aging-related changes. Since we found that a low-dose combination of fluvastatin and valsartan improved arterial wall characteristics, we aimed to further explore the mechanism behind these beneficial effects. Thus, from the previously obtained samples, we assessed the expression of longevity genes to explore the potential rejuvenating effect of our approach. We found that the low-dose fluvastatin and valsartan combination increased the expression of the *SIRT1* (1.8-fold; *p* < 0.0001), *PRKAA* (1.5-fold; *p* = 0.0262) and *KLOTHO* (1.7-fold; *p* < 0.0001) genes after 30 days, whereas no differences in the expression of the *NFE2L2*, *mTOR*, and *NF-κB* genes were observed. Fluvastatin or valsartan alone were less effective, increasing only the expression of the *SIRT1* gene to a lesser extent. Moreover, the expression of the *SIRT1* gene in the combination group positively correlated with telomerase activity and improvement of the arterial wall characteristics.

Importantly, the FDA recently approved the first interventional “anti-aging” study (MILES—Metformin in Longevity Study). Metformin, which, for this purpose, was repositioned from a solely antidiabetic drug to an “anti-aging” drug, is the interventional drug. This is based on a wealth of data indicating that metformin could influence the aging process, or more importantly, aging-related disorders. Interestingly, one of the most prominent of the several hypotheses underlying the “anti-aging” effects of metformin is the activation of longevity genes with consequent effects on energy metabolism, inflammation and oxidative stress. Several other currently ongoing studies with metformin are focusing on its “anti-aging” effects, such as VA-IMPACT, TAME and ePREDICE. In any event, a new period in which specific treatments of “aging-related disorders” are being studied, has already begun.

Sirtuins are a family of nicotinamide adenine dinucleotide (NAD)-dependent deacetylases, and according to some studies, are one of the key molecules involved in the regulation of aging and aging-related disorders [12]. *SIRT1* regulates DNA transcription and repair as well as cell survival, thus also inducing longevity. It was shown to have an important role in aging-related disorders of the cardiovascular [5] and nervous systems [13]. Some statins in therapeutic doses were shown to induce *SIRT1* expression, for example, simvastatin in endothelial progenitor cells [14]. On the other hand, atorvastatin and rosuvastatin reduced its expression in patients with coronary artery disease [15]. Until now, the effect of fluvastatin in therapeutic or low doses on *SIRT1* expression has not been assessed. To the best of our knowledge and according to the literature, no studies have assessed the effect of valsartan on the *SIRT1* gene in humans, either. A few studies were performed on rats or mice, in which valsartan and other sartans increased the expression of the *SIRT1* gene [16,17,18]. The *PRKAA* gene encodes the catalytic subunit of the AMPK. AMPK is the primary regulator of cellular responses and acts as a sensor to maintain energy balance within the cell [19]. In animal and cell culture studies, several statins were shown to induce the AMPK and eNOS pathway, acting in a vasoprotective manner [20,21,22]. Valsartan was also shown to act protectively through activation of the AMPK pathway in diabetic rats [23]; similar effects have been shown for telmisartan in human coronary artery cells [24]. The consequences of AMPK activations were divergent: In acute activation, it caused cell protection, whereas in chronic activation, it might have activated the pro-aging pathways and progressive degeneration during cellular senescence. There are various interactions between sirtuin and AMPK pathways [19]. The expression of *KLOTHO* decreased in aging-related disorders [25]. Valsartan in therapeutic doses increased the amount of plasma-soluble *KLOTHO* and consequently induced cardiorenal beneficial effects in patients with diabetes mellitus and diabetic kidney disease [26]. The effects of valsartan in low doses have not yet been studied in such a setting. The potential beneficial effects of statins on *KLOTHO* expression was only shown in animal studies [27]. The *NFE2L2* gene encodes a transcription factor, which regulates the proteins involved in responding to injury and inflammation. According to some studies, enhancing *NFE2L2* activity may be beneficial in diabetic cardiomyopathy, mitochondrial dysfunction, and as an anti-aging agent, but further studies are needed [28]. Fluvastatin was shown to induce *NFE2L2* in the vascular smooth muscle cells [29,30]. Mammalian target of Rapamycin (mTOR) was shown to have an important role in cardiovascular diseases, oxidative stress and longevity [31]. In animal or cell studies, statins influenced the *mTOR*, which was proven for fluvastatin in rats [32] and for lovastatin in vascular smooth muscle cells [33]. In one study on rats, valsartan induced cardio protection against ischemic-reperfusion injury through the mTOR [34]. *NF-κB* gene expression mediated vascular and myocardial inflammation and was additionally associated with impaired endothelial function [35]. There is evidence that both statins and sartans could reduce the *NF-κB* gene activation in various animal and cell line models [36,37].

To the best of our knowledge, no study like the present one, has been published. In our review of the literature, we found several different studies that assessed the effects of statins or sartans on longevity genes, but most of those studies were performed either on cell lines or animals. Therefore, the present study is the first to show that our new preventive cardiovascular approach, which was proven to induce the improvement of arterial wall functional and structural characteristics, and consequently decrease arterial age, additionally acted through the expression of several longevity genes. This could be one of the mechanisms lying behind the beneficial effects observed in our previous clinical studies [7,8,9]. Nevertheless, one of the limitations of the present study is that we used only qPCR, but validation by Western blotting would be of added value.

The results of the present study indicate that our innovative approach using short-term low-dose fluvastatin and valsartan has a potential in inducing the expression of certain longevity genes. These effects could be anti-aging or rejuvenating as well as act as a potential specific prevention/treatment for “aging-related” disorders. It can be speculated that using cycling, intermittent treatment with low-dose combination (every 6–12 years) starting at middle-age could postpone the occurrence of aging-related disorders. On the other hand, this approach could be used in the same population and with the same aim as metformin in the MILES trial. One of the major advantages of this approach is its cyclic, intermittent character, which, as previously described, could potentially activate the beneficial longevity genes for a time short enough not activate their contra regulatory mechanisms as well. With intermittently repeating cycles, this could lead to repetitive beneficial activations of the protective longevity genes. Thus, it could be speculated that the cumulative effect of these repeating cycles of treatment might eventually lead to successful specific prevention/treatment of “aging-related” disorders, most likely cardiovascular “aging-related” disorders.

## 4. Materials and Methods

### 4.1. Participants and Study Design

The stored blood samples from our three prior studies were used together for the present longevity gene expression study. Overall, 130 middle-aged, apparently healthy male participants were recruited and treated for one month (30 days): 25 persons with fluvastatin 10 mg daily, 20 persons with valsartan 20 mg daily, and 20 persons with a low-dose combination of fluvastatin (10 mg daily) and valsartan (20 mg daily). Accordingly, 65 participants received placebo. All the participants were blindly randomized into the relevant group, as in our previous studies, which are described in more detail elsewhere [7,8,9]. Blood samples were collected and ultrasound measurements of the arterial wall properties (endothelial function, arterial stiffness) were performed at the beginning and at the end of the treatment period (day 0 and day 30). The measurements were also repeated five months after treatment discontinuation.

The National Medical Ethics Committee of Slovenia approved the studies (Approval date 3 July 2009, Approval No.: 21k/05/09) and informed consent was obtained from all participants. Inclusion criteria were: age between 30 and 50 years, non-smoking status, normal blood pressure values, body mass index values below 30 kg/m^2^, no clinical cardiovascular disease, no history of any other chronic disease, and no regular medication therapy. The characteristics of the subjects were already extensively described in our previous publications [7,8,9].

### 4.2. Blood Sampling

Three samples of whole peripheral blood were collected from each participant: before treatment (day 0), after treatment conclusion (day 30), and five months after treatment discontinuation (follow-up). The whole blood samples were collected in 10 mL EDTA tubes and stored at −80 °C. Prior to RNA extraction, samples were centrifuged at 4000 rpm for 25 min to obtain the pellet of cells and cell debris. The pellets were then used for RNA extraction.

### 4.3. RNA Isolation

Total RNA was isolated using a miRNeasy Mini kit (Qiagen, Hilden, Germany) according to the manufacturer’s instructions. RNA was quantified using the NanoDrop, and cDNA was synthesized from 300 ng of the total RNA using the High-Capacity cDNA Reverse Transcription Kit with RNase Inhibitor (Applied Biosystems, Foster City, CA, USA) according to the manufacturer’s protocol.

### 4.4. Quantitative Real-Time PCR (qPCR) for Human Telomerase Reverse Transcriptase (hTERT) Expression

The expression of target genes in the tested samples was performed using TaqMan Gene Expression Assays (Applied Biosystems) according to the manufacturer’s instructions: Assay Hs00183100_m1 for the *KLOTHO* gene; assay Hs01009006_m1 for the Sirtuin 1 (*SIRT1*) gene; assay Hs01562315_m1 for the 5′AMP-activated protein kinase (*AMPK*) gene; assay Hs00765730_m1 for the Nuclear factor κB (*NF-κB1*) gene; assay Hs00234522_m1 for the Mechanistic target of rapamycin (*mTOR*) gene and assay Hs00975961_g1 for the Nuclear factor (erythroid-derived 2)-like 2 (*NFE2L2*) gene. The housekeeping gene glyceraldehyde 3-phoshate dehydrogenase (*GAPDH*) was used as an endogenous control. Described briefly, qPCR was performed using the ABI 7900 instrument (Applied Biosystems). Individual qPCR reactions were carried out in 10 µL reaction mix with 2xTaqMan Universal PCR Master Mix (Applied Biosystems), 1× TaqMan Gene Expression Assay (Applied Biosystems) and 200 ng cDNA. Each sample was analyzed in triplicate. RNA isolated from healthy volunteers (*n* = 5) was used as a positive control for target genes expression. In each run, the dilutions of control RNA (pool of RNA from healthy volunteers) was included. The data were analyzed by the SDS2.4 software and *C*t values were extracted. Fold-differences in *hTERT* expression were calculated using the comparative Ct method as described previously [38], where data were normalized to day 0 for each participant.

### 4.5. Data Analysis

All values were expressed as means ± SEM. Differences between values were assessed by one-way analysis of variance (ANOVA). When significant interaction was present, the Bonferroni post-test was performed. Benjamini-Hochberg’s correction method was used to control false discovery rate (FDR), with significance threshold set at *p* < 0.05. Correlations between arterial wall properties and telomerase activity that were described previously [7,8,9,11] and longevity genes expression assessed in the present study were calculated after 30 days treatment period using Pearson correlation coefficients. A *p* < 0.05 was considered significant. All statistical analyses were performed using the Graph Pad Prism 5.0 software.

## 5. Conclusions

In conclusion, the present study has shown that low-dose fluvastatin and valsartan treatment increased the expression of beneficial longevity genes (*SIRT1*, *PRKAA*, and *KLOTHO*) and could therefore represent a promising new treatment approach for “aging-related” disorders. Additional, population-based research is needed to additionally prove the proposed concept.

## Abbreviations

AMPK	AMP-activated protein kinase
ANOVA	Analysis of variance
COMB	Combination group
CTRL	Placebo group
FDA	Food and Drug Administration
FLU	Fluvastatin group
FMD	Flow mediated dilation
*hTERT*	human telomerase reverse transcriptase
MILES	Metformin in Longevity Study
mTOR	Mechanistic target of rapamycin
NAD	Nicotinamide adenine dinucleotide
NF-κB1	Nuclear factor κB
*NFE2L2*	Nuclear factor (erythroid-derived 2)-like 2 gene
*PRKAA*	5′-AMP-activated protein kinase catalytic subunit α-2 gene
PWV	Pulse wave velocity
qPCR	Quantitative real-time polymerase chain reaction
*SIRT1*	Sirtuin 1 gene
VAL	Valsartan group
